# Enzymatic and Microwave Pretreatments and Supercritical CO_2_ Extraction for Improving Extraction Efficiency and Quality of *Origanum vulgare* L. spp. *hirtum* Extracts

**DOI:** 10.3390/plants11010054

**Published:** 2021-12-25

**Authors:** Jelena Vladić, Ana Rita C. Duarte, Sanja Radman, Siniša Simić, Igor Jerković

**Affiliations:** 1 Faculdade de Ciências e Tecnologia, Universidade Nova de Lisboa, 2829-516 Caparica, Portugal; ard08968@fct.unl.pt; 2 Faculty of Technology, University of Novi Sad, Bulevar cara Lazara 1, 21000 Novi Sad, Serbia; wsimic177@gmail.com; 3 Faculty of Chemistry and Technology, University of Split, Ruđera Boškovića 35, 21000 Split, Croatia; sradman@ktf-split.hr (S.R.); igor@ktf-split.hr (I.J.)

**Keywords:** *Origanum vulgare*, supercritical carbon dioxide, microwave pretreatment, enzymatic pretreatment

## Abstract

The goal of the study was to establish a procedure for improving the efficiency of supercritical carbon dioxide (scCO_2_) extraction of *Origanum vulgare* L. spp. *hirtum* (Greek oregano) and enhancing the quality of obtained extracts. Microwave and enzymatic pretreatments of the plant material were applied prior to the scCO_2_ extraction. It was determined that the microwave pretreatment with irradiation power 360 W during 2 min accelerated the extraction of lipophilic compounds and provided a twofold higher extraction yield compared to the control. Moreover, this pretreatment also led to an increase in oxygenated monoterpenes content and the most dominant component carvacrol, as well as the extracts’ antioxidant activity. The enzymatic pretreatment caused a significant increase in the extraction yield and the attainment of the extract with the most potent antioxidant properties. Coupling the pretreatments with scCO_2_ extraction improves the process of obtaining high value lipophilic products of oregano in terms of utilization of the plant material, acceleration of the extraction with the possibility to adjust its selectivity and quality of extracts, and enhancement of biological activity.

## 1. Introduction

Aromatic medicinal plant oregano (*Origanum vulgare* L.) belongs to the family Lamiaceae, genus Origanum, which economically represents one of the most important genera of this family [1]. Oregano represents a highly important natural resource widely used as a fragrance in the cosmetic and perfume industry. Additionally, it is one of the most common flavors used in different cuisines for taste improvement and shelf-life extension [2]. In addition to being a food, flavor, and cosmetic ingredient, medicinal application of oregano and its compounds is highly significant and widespread due to its antimicrobial, antioxidant, anticancer, and anti-inflammatory activities [3,4,5,6,7,8]. These properties are mainly attributed to the presence of essential oil and its volatile aromatic constituents. Chemical aromatic profile and content of oregano vary significantly depending on the subspecies, climate, geographic position, development phase, way of picking, processing, storage, and other factors [9]. An advantage of the oregano species is that it can be grown in different environments. Hence, Greek oregano is cultivated successfully in temperate climate [10]. Also, this herbal species can adapt to more extreme environmental conditions [11]. Oregano essential oil can be classified into two main chemotypes: (a) containing thymol, carvacrol, or both as the major components and (b) containing germacrene D and terpinen-4-ol [5].

Supercritical carbon dioxide (scCO_2_) extraction was established as a superior approach for obtaining aromatic lipophilic compounds from natural resources, compared to conventional methods such as hydrodistillation, steam distillation, and solvent extraction. The characteristics of this extraction process are the application of safe solvents, moderate temperatures which avoid degradation of thermolabile compounds, possibility of adjusting selectivity, better exploitation of resources, and enhanced extraction efficiency [12]. Moreover, extracts obtained by scCO_2_ extraction retained a much better aroma than steam distilled extracts [13]. Carbon dioxide in supercritical state was applied as an extraction solvent for aromatic volatile compounds of oregano in several studies [14,15]. Busata et al. [16] demonstrated that scCO_2_ provides a higher yield compared to that of hydrodistillation. Also, Leeke et al. [17] improved the efficiency of extraction of *Origanum vulgare* L. ssp. *virens* (Hoffm. et Link) *letswarrt* using scCO_2_ in the presence of H_2_O. Pérez et al. and Menaker et al.’s studies determined that the addition of ethanol improves the oregano extraction yield significantly [18,19].

Furthermore, the increase in the recovery of the volatile constituents from herbal material can be achieved by pretreating the herbal material. Pretreatments weaken the cellular wall and decrease the internal resistances of the material which increases the availability of the target components to the solvent [15]. In this way, the extraction can be accelerated, and more efficient material exploitation can be achieved. However, it is important that the pretreatment does not degrade the quality of the obtained product in terms of chemical composition or biological activity. Therefore, depending on the material’s characteristics and target components, different pretreatments can be used including physical, enzymatic, biological, and chemical pretreatments. 

Thermal pretreatments or treatments where the temperature is increased during the process can lead to the degradation of components, hence their optimization is necessary to avoid degradation. Mechanical pretreatments of plant materials are adequate for materials with a compact cellular wall. Moreover, these materials require a significant disruption in the material structure and decrease in particle size, such as the *Nannochloropsis oculate* algae and *Usnea barbata* lichen [20,21]. Nevertheless, these treatments can cause excessive loss of components of interest, especially volatile compounds. In addition, soaking the material in H_2_O and ethanol can soften the cellular structure and facilitate the solvent penetration, but it is time-consuming [22]. The application of microwave radiation is a process in which energy is distributed directly to the plant material causing molecular interaction with the electromagnetic field. In that way, it achieves uniform heating in a very short time. This increases the porosity of the material and facilitates the penetration of the solvent and the compounds availability [23]. Considering that microwave radiation includes the emission of energy which is mostly absorbed by the plant moisture content, combined exposure of the material to moistening and microwaves can achieve a significant increase in permeability of the material in a short time [24]. Enzymatic pretreatment is a process which disrupts the cellular wall through biodegradation in mild conditions. This pretreatment can achieve a higher release of the oil from the plant [25]. The additional advantage of the enzymatic pretreatment is that it does not require complex equipment [26].

Thus far, oregano pretreatments were not investigated. Given the importance of oregano lipophilic bioactive components and their high applicability and demand, there is a constant need for improving methodology for their recovery. Therefore, this study evaluated the procedures for the improvement of the extraction efficiency of the oregano lipophilic fraction. Furthermore, the impact of microwave and enzymatic pretreatments of the plant material on the extraction yield and chemical profile of the obtained extracts was investigated. The microwave pretreatment was applied with different irradiation powers on the materials soaked in H_2_O and dry materials. In addition, the antioxidant activity of extracts was investigated.

## 2. Results and Discussion

### 2.1. Extraction Yield

To determine the adequacy of the pretreatment in terms of efficiency and quality of obtained extracts, all extractions of differently pretreated materials and control (without pretreatment) were conducted at identical conditions (200 bar and 40 °C). The extractions were conducted at 40 °C to avoid volatilization and decomposition of thermally unstable compounds. Moreover, the 200 bar pressure was selected based on the Rodrigues et al. study [14] where it was determined that the application of this pressure value and 40 °C temperature provide a two-fold higher extraction yield compared to 100 bar pressure and 40 °C temperature extraction conditions. Furthermore, the 200 bar pressure was selected to decrease the extraction of waxes, which are coextracted at higher pressures [27]. 

**Figure 1:**
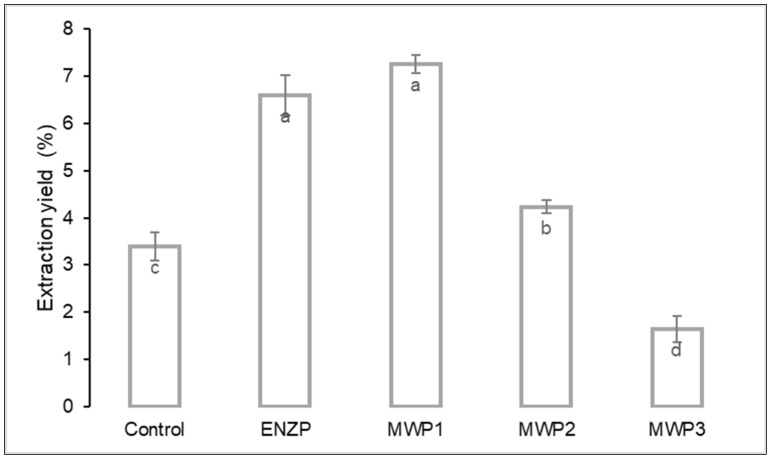
Total extraction yield of supercritical carbon dioxide extraction (pressure 200 bar, temperature 40 °C, extraction time 4 h) of pretreated samples and control. Different letters (a, b, c, d) indicate significant difference between samples (*p <* 0.05).

**Figure 2:**
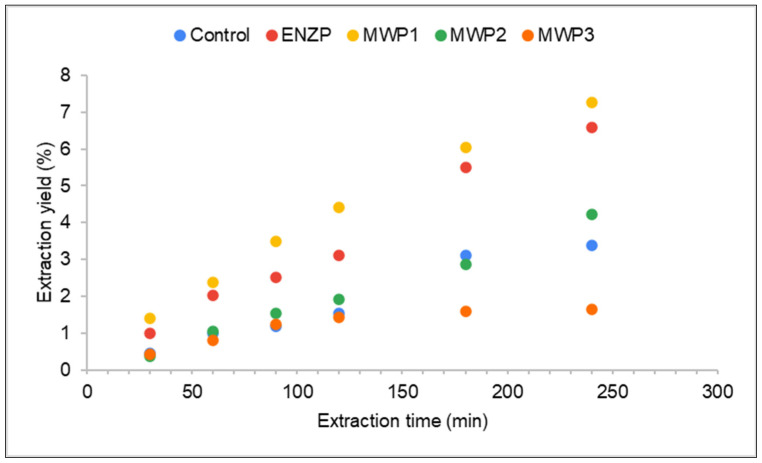
Supercritical carbon dioxide extraction kinetics of oregano (pressure 200 bar, temperature 40 °C, extraction time 4 h).

The yield of the scCO_2_ extraction (g of obtained extract per 100 g of dry herbal material) of the control sample was 3.39% (Figure 1 and Figure 2). The yields of oregano scCO_2_ extractions vary significantly in different studies depending on the origin, subspecies, and characteristics of the raw material and scCO_2_ extraction conditions. The study Rodrigues et al. [14] used the same scCO_2_ extraction temperature and pressure (40 °C and 200 bar) to extract commercial oregano and achieved the yield of 1.32% ± 0.03, while Busatta et al. [16] extracted the leaves of oregano from Chile and gained the yield of 1.75% ± 0.25. Moreover, Mechergui et al. [1] reported the yield of 2.23 and 2.07% (v/w) at 40 °C and 90 bar for Tunisian *O. glandulosum*. Furthermore, using 350 bar pressure and 40 °C temperature achieved the yield of 4.89% [28] and 4.77% at 300 bar and 40 °C [27]. 

In oregano as well as in Lamiaceae species, the essential oil is found in the multicellular epidermal glands, glandular trichomes [29]. The pretreatments have the aim to disrupt physical barriers, namely, the trichomes wall, and in that way increase the availability and release of lipophilic compounds. After pretreating the plant material, total scCO_2_ extraction yields obtained were in the range of 1.65 to 7.25%. Figure 1 shows how the total extraction yield changed after the pretreatment compared to the control. Treating the material intensified the intraparticle transport [30]. The highest total yield (7.25%) was achieved by extracting the herbal material after MWP1 pretreatment (2 min, 360 W), which is 2.14 times higher yield compared to the control extraction (3.39%). Higher irradiation power (800 W) also resulted in an increase in the recovery of lipophilic compounds compared to the control. However, the total yield was significantly lower (4.23%) compared to MWP1. The benefit of microwave pretreatment is that its energy, due to the interaction between the plant material and the electromagnetic field, is distributed to the plant material directly. Hence, during the process, uniform heating of the material is achieved leading to extraction improvement [31]. On the other hand, due to exposure to microwaves at excessively high irradiation powers, there is a rapid increase in temperature. Therefore, loss of components, especially volatile ones, can occur.

For the microwaves to exhibit a positive effect on the extraction efficiency, moisture in the material is necessary. Due to water absorption, heating occurs, and herbal structures have the role of an ionic conductor leading to accelerated hydrolysis [32]. Considering that dry oregano material contained the moisture content of approximately 10%, it might be insufficient for an adequate interaction with the microwaves. Furthermore, overheating of the plant material might have occurred due to its exposure to radiation and possible evaporation or degradation of components in MWP3. Therefore, exposing the dry plant material to microwave radiation (MWP3) resulted in a significantly decreased total yield (approximately 2 times less than the control). The lower irradiation power (360 W) of the hydrated oregano material for 2 min represented a more efficient pretreatment.

Microwave pretreatment of pomegranate seed [33] and rapeseed [34] was used without the addition of H_2_O. However, the location of oil and structure of the plant material is significantly different compared to oregano leaves, therefore, the dry material irradiation was beneficial. Dry seeds contain traces of moisture which heats up, evaporates, and causes high pressure on the cell membrane when exposed to microwave radiation. Due to the formed pressure within the seeds, the structure weakens and consequently, there is an increase in oil release. In addition, seed oil has a significantly different chemical composition and contains fewer volatile components [32].

The enzymatic pretreatment was conducted previously with a commercial mixture of enzymes containing a wide range of carbohydrates. The application of the enzymatic pretreatment led to a significant, almost twofold increase in the extraction yield (6.59% compared to the control 3.39%). Exposing the plant material to cellular wall degradation enzymes resulted in damage and hydrolysis of the material’s structures and consequently, facilitation of the solvent’s penetration and components’ availability, hence their faster and more efficient release. The positive impact of the enzymatic activity as the pretreatment prior to volatile oil separation was recorded in obtaining celery volatile oil, where the yield increased by 22–27% [35]; garlic with a twofold oil yield increase [36], and cumin with 18–22% oil yield increase [37]. The cocktail of cell wall degrading enzymes (cellulase, protease, xylanase, and pectinase) resulted in an increase in the grape seed oil yield by 44% [38]. Basil leaves were pretreated with hemicellulase, cellulase, and viscozyme. The viscozyme pretreatment was the most effective for obtaining the essential oil by hydrodistillation and caused changes in the morphology of glandular trichomes. After the enzymatic treatment, the number of deflated glandular trichomes increased [39]. Also, the yields of *Laurus nobilis* L., *Thymus capitatus* L., and *Rosmarinus officinalis* L. essential oils obtained by hydrodistillation was improved significantly by applying various enzymatic pretreatments [40,41]. Enzymatic pretreatment of ginger improved the extraction of oleoresin. Additionally, the treatment with viscozyme and α-amylase followed by acetone extraction was the most efficient for the extraction of the major bioactive constituent 6-gingerol [42].

The confirmation of the effectiveness of the application of pretreatment MWP1 and ENZP can be achieved by comparing the time necessary for the recovery of the components. In the first 30 min of the extraction, the extraction yield with ENZP and MWP1 was twice as high compared to the control (Figure 2). After 90 min of extraction of the MWP1 sample, the same total yield was achieved as for the control after 4 h of extraction. Therefore, the plant material pretreatment intensified mass transfer and decreased the time required for the recovery of lipophilic components.

The extraction time of 4 h was selected based on the control extraction where it was determined that after 4 h of extraction, there was no significant yield increase. Therefore, Figure 2 shows all extractions with extraction time of 4 h for better comparison. However, it is evident that ENZP, MWP1, and MWP2 extractions had a rising trajectory even after 4 h of extraction. For this reason, the kinetics of the extractions were monitored every 30 min after the initial 4 h. It was determined that the exhaustion of the plant material MWP2 was achieved after the additional 2.5 h of extraction (total 6.5 h; total extraction yield 5.35%), while the ENZP and MWP1 extractions were completed after additional 1.5 h (total extraction time 5.5 h; ENZP total extraction yield 7.09% and MWP1 total extraction yield 7.92%). The long extraction time represents a drawback to the implementation of these processes on an industrial level. In addition to the application of the pretreatment that improves the extraction yield significantly, it is necessary to optimize the extraction parameters such as the CO_2_ flow and pressure to determine the optimal extraction conditions.

### 2.2. Chemical Profile

**Figure 3:**
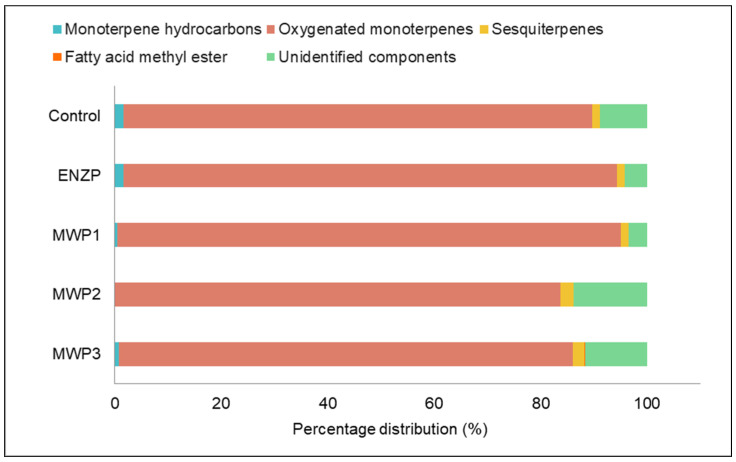
Percentage distribution of groups of aromatic compounds in oregano extracts obtained by supercritical carbon dioxide extraction (pressure 200 bar, temperature 40 °C, extraction time 4 h).

Increasing the extraction yield by using microwave and enzymatic pretreatments represents highly important economic parameter. In addition, by increasing the yield, more efficient exploitation of the plant material is achieved, which is also one of the sustainable goals of the extraction processes. Pretreatment can also impact the quality of the obtained extracts in terms of their chemical profile and biological activities. The process of obtaining the final extract needs to be optimal, that is to achieve an increase in extraction yield and optimal exploitation of the material without affecting quality. For this reason, the obtained extracts were investigated by GC/MS (Table 1, Figure 3).

In all samples, oxygenated monoterpenes were the most dominant group of compounds with 83.7–94.67%. Besides oxygenated monoterpenes, the components that belong to the group of monoterpene hydrocarbons and sesquiterpenes were identified at lower abundance. Such distribution among component groups with dominant monoterpene and sesquiterpene hydrocarbons up to 2–3% is characteristic of *O. vulgare* L. subsp. *hirtum* [10]. MWP1 and ENZP led to an increase of oxygenated compounds abundance in the extracts (92.69% and 94.67%, respectively; there were no statistically significant differences among these groups) compared to the control (88.06%) (Figure 3). This is important considering that oxygenated compounds are more odoriferous than monoterpene hydrocarbons, and the odor and taste represent good indicators of the essential oil quality [43]. An increase in oxygenated monoterpenes was recorded in hydrodistillated essential oil from bay leaves after the enzyme pretreatement [40] and in the essential oil from *Fructus forsythia* obtained through enzyme-assisted microwave hydrodistillation [44]. 

In terms of qualitative difference in the aromatic profile of extracts, the biggest difference compared to the control extract was in MWP2 extract in which the number of identified components was decreased twofold compared to that of the control. Due to excessive irradiation power and heating of the plant material, it is possible that the evaporation or decomposition of compounds occurred. Hence in MWP2 sample obtained with the pretreatment at higher irradiation power, no components were identified from the monoterpene hydrocarbons group, nor sabinene hydrate, borneol, thymol methyl ether, and carvacrol methyl ether from the group of oxygenated monoterpenes. Probably due to more intensive heating in MWP2 and MWP3, there was a loss in the most volatile compounds, monoterpenes, and consequently an increase in the abundance of less temperature-sensitive sesquiterpenes (Table 1 and Figure 3). Additionally, the percentage of unidentified components in these two samples increased, therefore, the total peak area of identified components decreased (86.21 and 88.38% for MWP2 and MWP3, respectively) compared to the control sample. The absence of monoterpene hydrocarbons due to the exposure to microwaves was recorded in a study in which hydrodistillation and microwave-assisted hydrodistillation of *Ocimum basilicum* L. were compared. Furthermore, in the essential oil obtained by microwave-assisted hydrodistillation there were no monoterpene hydrocarbons, although their presence was determined in the essential oil obtained by hydrodistillation. The authors attributed this absence of monoterpene hydrocarbons to their possible oxidation [45].

The most dominant constituent of the aromatic profiles was carvacrol with over 70%. Moreover, the dominance of carvacrol indicated carvacrol-type of chemotype of oregano. As previously mentioned, the chemical composition of oregano varies significantly in different studies due to the differences in geographical and climate conditions, making comparison difficult. *O. vulgare* subsp. *hirtum* from the southern part of Greece contained 57.4–69.6% carvacrol, while the oregano plant material from the northern part of the country had a significantly higher thymol content [46]. Apart from the main chemotypes, there are intermediary chemotypes (thymol/carvacrol and carvacrol/thymol) [9]. *O. vulgare* from Chile and *O. majorana* from Egypt had a similar chemical composition with the most dominant component terpinen-4-ol in the essential oil obtained by hydrodistillation and *cis*-sabinene hydrate by scCO_2_ [16]. Oregano (*O. vulgare*) cultivated in Estonia had thymol and carvacrol as the most dominant constituents of oregano scCO_2_ extract [18]. In extracts obtained at 35 °C and 200 bar, carvacrol (86.9%) and thymol (11.3%) were present [47]. 

Using a commercial oregano sample from Mexico and extraction parameters 40–60 °C, 100–300 bar, and 4–8 g/min co-solvent, extracts with up to 30% carvacrol, heneicosane (6.99–8.21%), nonacosane (7.22–11.78%), docosane (4.18–7.18%), borneol (3.65–4.35%), and thymol (3.97–4.51%) were obtained [19]. Santoyo et al. [5] applied different extraction conditions (40, 60 °C, 150–350 bar; 0–7 g∙min^−1^ of ethanol as cosolvent) and obtained oregano extracts with 49.43% of carvacrol and 25.11% of *trans*-sabinene.

Different oregano chemotypes exhibit various biological activities, however, carvacrol chemotype is the most important one in terms of medicinal application [10]. The dominant group of components of Greek oregano was the monoterpenes group with the highest content of carvacrol (64.44–73.85%) in essential oil [10]. The content of carvacrol was in accordance with the studies that investigated the chemical composition of Greek oregano [10,48,49,50]. The control extract contained 74.89% of carvacrol. The pretreatments led to an increase in carvacrol abundance in MWP1, MWP2, and ENZP samples compared to the control. In the extract obtained after MWP1 pretreatment, the presence of carvacrol was the highest (80.44%). Additionally, the extracts obtained after MWP2 and ENZP contained 76.94% and 77.17% of carvacrol, respectively. The beneficial impact of the enzymatic pretreatment on the recovery of carvacrol was observed in the attainment of thyme essential oil [41]. Negative impact or decrease in the presence of carvacrol compared to the control extract was found in MWP3 sample (70.95%) and it was potentially provoked by excessive heating of the plant material. 

Carvacrol represents a commercially very significant monoterpene. Its antimicrobial potential was confirmed by numerous studies against different human, foodborne, and fungal plant pathogens [51,52,53]. According to Ben Srfa et al. [54], carvacrol’s hydrophobic properties and free hydroxyl group are responsible for its antimicrobial mechanism. In addition, its numerous biological activities were confirmed including antimicrobial, antitumor, antimutagenic, antigenotoxic, analgesic, antispasmodic, anti-inflammatory, angiogenic, antiparasitic, antiplatelet, AChe inhibitory, antielastase, insecticidal, and hepatoprotective [50].

Thymol, the isomer of carvacrol and a pharmacologically important monoterpene, was present from 2.26% to 2.47% and the pretreatments did not impact its presence in the extracts significantly. Both phenolic monoterpenes represent approved food additives due to their potency against Gram-positive and Gram-negative bacteria [55]. Also, it was demonstrated that they can act in synergy with synthetic antibiotics and in that way act against bacteria resistance [56]. According to the European Pharmacopoeia, the parameter of quality of the oregano oil is the content of carvacrol and thymol of minimum 60%.

Minor compounds which were detected in the extracts are also biologically active compounds applied in different areas and industries. Biochemical precursors of carvacrol and thymol, *γ*-terpinene and *p*-cymene, were present in the extracts under 1%. Although in low concentrations, their presence is significant considering that the biological activity does not depend on the most dominant component solely but is rather a result of the synergy of all its constituents [43]. It was determined that the antimicrobial activity of carvacrol can be improved by the presence of its precursor *p*-cymene. This aromatic hydrocarbon exhibits a range of activities compared to carvacrol; however, *p*-cymene causes the swelling of the cytoplasmatic membrane to a higher extent than carvacrol. Therefore, in combination with carvacrol, *p*-cymene is incorporated in the cytoplasmatic membrane and leads to a more intensive transport of carvacrol through the membrane [57]. Additionally, *p*-cymene and *γ*-terpinene also exhibit antioxidant and antibacterial activity and represent commercially significant components used in the production of perfumes and aromas [58].

The presence of other important fragrance compounds was also detected, including *α*-terpineol and its isomer terpinen-4-ol. The percentage of terpinen-4-ol was decreased by applying the pretreatments. *α*-Terpineol in the control extract was not identified, but it was present in all other samples. Apart from being used as a fragrance, this monoterpene alcohol has medicinal importance with numerous biological activities like anticancer, cardioprotective, antiulcer, and antinociceptive. It also improves the chemical skin penetration for pharmaceutical formulations [59].

Sabinene hydrate monoterpene was characterized as an adequate alternative to synthetic antioxidants for preventing peroxide formation during storage and it was more efficient than carvacrol and thymol in terms of preservation [60]. Moreover, due to its antioxidant properties, carvacrol methyl ether can also be an alternative to synthetic antioxidants in preserving and improving the stability of foods like sunflower oil during the deep-frying process [61].

Bicyclic sesquiterpene and naturally occurring cannabinoid ligand *trans*-caryophyllene is a constituent of the volatile profile of oregano with anti-inflammatory, antiviral, immunomodulatory, anticancer, and analgesic activities. Also, a recent study suggests that *β*-caryophyllene and the plants containing *β*-caryophyllene as a major constituent may be candidates for developing antiviral and immunomodulatory therapies for coronaviruses [62]. Its metabolite, caryophyllene oxide, is also attributed with anticancer and analgesic properties. Because of their preservation properties, they are used as natural cosmetics and food additives approved as flavorings by the Food and Drug Administration (FDA) and by the European Food Safety Authority (EFSA) [63]. *trans*-Caryophyllene received additional attention because it belongs to the class of phytocannabinoids. The advantage of this cannabinoid is that it does not show an affinity for receptor type 1 associated with psychoactive side effects (CB_1_), and its application activates only type 2 receptor, classifying it into safe natural cannabinoids [63]. Compared to that of the control extracts, the content of *trans*-caryophyllene was increased after all pretreatments except for MWP1. Another tricyclic sesquiterpene alcohol spathulenol, identified in all samples, demonstrated antioxidant, anti-inflammatory, antiproliferative, and antimycobacterial activities [64]. Fatty acid methyl ester, methyl palmitate, and *β*-bisabolene were present only in the MWP3 extract.

Supercritical carbon dioxide is a solvent that provides the adjustment of selectivity of extraction towards target components by varying process parameters. Considering that the pretreatments show selectivity towards specific individual components, an approach which integrates a pretreatment followed by scCO_2_ can increase the selectivity towards components of interest with optimal exploitation of the plant material, increased efficiency, and decreased process time.

To implement these processes at industrial scale, a detailed scale up study is necessary to achieve economic feasibility. The enzymatic pretreatment includes costs of enzyme production [65], while the scale-up of the microwave pretreatment requires a careful design of the equipment to provide a safe and reliable process [66,67].

### 2.3. Antioxidant Activity

The obtained extracts were investigated in terms of antioxidant activity to determine the impact of the pretreatment. DPPH test was used for determining the antioxidant activity since it is a fast, simple, and reproducible assay. DPPH radical is stable, and it is not necessary to generate it as in other tests. Therefore, DPPH test is widely applied for the evaluation of the antioxidant activity of the samples obtained from natural resources [68].

**Figure 4:**
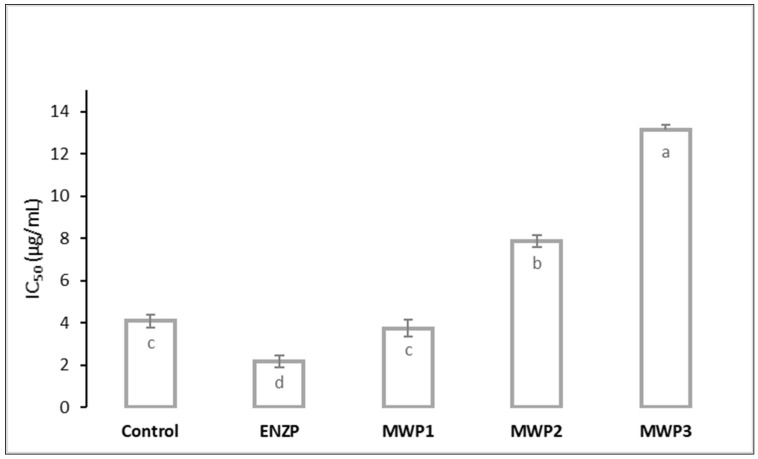
Antioxidant activity (expressed as IC_50_ value) of oregano extracts obtained by supercritical carbon dioxide extraction (pressure 200 bar, temperature 40 °C, extraction time 4 h). Different letters (a, b, c, d) indicate significant difference between samples (*p* < 0.05).

The significant potential of oregano extracts obtained by scCO_2_ was determined in previous studies. Žitek et al. [6] applied temperatures 40 °C and 60 °C and pressures 150 and 250 bar and determined that the scCO_2_ extract obtained at 250 bar and 60 °C extraction conditions exhibited the most significant antioxidant activity similar to the one of the extracts obtained by water maceration. However, the authors emphasized the advantage of using scCO_2_ because optimal maceration temperature was considerably higher (83 °C). The scCO_2_ Greek oregano extract showed a higher antioxidant activity than essential oil obtained by hydrodistillation [48]. Moreover, the antioxidant activity of oregano essential oil and extracts is attributed to their dominant components thymol and carvacrol, whose antioxidant activity was confirmed by numerous studies. It was determined that thymol and carvacrol can exhibit individual and joint activity, which reduces oxidative stress, prevents lipid peroxidation, and stimulates the activity of endogenous antioxidative enzymes [69]. Furthermore, Kulišić et al. [70] stated that antioxidant activity of oregano essential oil is the result of the presence of the dominant monoterpenic phenols, but it can also occur due to the synergistic effect of the components that have oxygen. Extracts MWP2 and MWP3, which had the lowest antioxidant activity, had a lower content of oxygenated monoterpenes and a higher content of sesquterpenes. According to Serrano et al. [71], sesquterpenes can exhibit a lower antioxidant activity. The most significant improvement of antioxidant properties compared to the control was determined in the enzymatically pretreated extract, followed by MWP1 (Figure 4). Other pretreatments led to a decrease in the extracts’ antioxidant activity. Antioxidant activity of all scCO_2_ extracts was higher than the antioxidant activity of oregano essential oils obtained by hydrodistillation (0.0542 mg/mL) and microwave-assisted hydrodistillation (0.0604 mg/mL) [45].

The correlation between the content of carvacrol and antioxidant activity was not observed. A similar pattern was noticed in the study where *Satureja montana* L. was subjected to ultrasound (ultrasonic power 125 W and frequency 50 Hz) and high pressure (100 bar and 40 °C) pretreatments. Although after the pretreatment carvacrol content was increased by 25%, the total antioxidant response of the extracts was lowered [22]. Additionally, in in vivo studies where *S. montana* extracts with significantly different content of extracts were administered, it was observed that antioxidant properties of extracts with a higher content of carvacrol were not pronounced [72,73]. Therefore, carvacrol is not the main carrier of antioxidant activity of the extracts, but the properties of the extracts are a consequence of possible synergistic and antagonistic effects of all constituents. The enzyme pretreatment enhanced the antioxidant activity of the bay leaves essential oil; however, the antioxidant activity of essential oil is a consequence of the interaction between different compounds of the essential oil, and it is not solely related to its major component 1,8-cineole [40]. 

## 3. Materials and Methods

### 3.1. Plant Material and Chemicals

*O. vulgare* spp. *hirtum* aerial parts were commercial samples purchased in the supermarket Diellas Markets (Corfu, Greece). The mean particle size (0.26 mm) of the material was determined using vibration sieve sets (CISA, Cedaceria, Spain). The carbon dioxide (Messer, Novi Sad, Serbia) with purity >99.98% (w/w) was used for the lab-scale scCO_2_ extraction. Viscozyme, a cellulolytic enzyme mixture (which contains a wide range of carbohydrases, including arabanase, cellulase, β-glucanase, hemicellulase, and xylanase), methanol, and 2,2-diphenyl-1-picrylhydrazyl (DPPH) were purchased from Sigma-Aldrich. 

### 3.2. Pretreatment of Material

Grounded oregano material (40 g per each pretreatment) was used in the pretreatments according to the following procedures:

Enzymatic pretreatment (*ENZP*)—mixing herbal material and enzymatic solution of Viscozyme (ratio herbal material/solution 1:5 (*m/V*)) and incubation at 45 °C for 60 min. Viscozyme solution was prepared with acetate buffer pH 4.9 at concentration 8% with respect to the plant material. 

Microwave pretreatment 1 (*MWP1*)—mixing herbal material and H_2_O in 1:5 (*m/V*) ratio and exposure to microwave irradiation power 360 W for 2 min (9 W/g). 

Microwave pretreatment 2 (*MWP2*)—mixing herbal material and H_2_O in 1:5 (*m/V*) ratio and exposure to microwave irradiation power 800 W for 2 min (20 W/g).

Microwave pretreatment 3 (*MWP3*)—exposure of dry material (without adding H_2_O) to microwave irradiation power 360 W for 2 min.

The microwave pretreatment was conducted using a microwave system consisting of a modified commercial microwave oven (NN-E201W, Panasonic, Osaka, Japan), appropriate glass apparatus with round flask, and condenser. Flask with material and H_2_O were placed into the oven and connected with the condenser. The time of the microwave pretreatment (2 min) was determined through preliminary research as a time during which essential oil separation did not occur. Moreover, this was established through microwave-assisted hydrodistillation conducted using the same microwave system with Clevenger apparatus. After the pretreatments, the plant material was separated from the liquid phase with vacuum filtration and dried in a freeze drier (Alpha 1-2 LPlus, Christ, Osterode am Harz, Germany). The dried material was used for the scCO_2_ extraction.

### 3.3. Supercritical Carbon Dioxide Extraction (scCO_2_)

The high-pressure extraction system (HPEP, NOVA-Swiss, Effretikon, Switzerland) was used for scCO_2_. The main specifications of the scCO_2_ unit were as follows: gas cylinder with CO_2_, diaphragm type compressor (with a pressure range up to 1000 bar), extractor vessel with heating jacket (internal volume 200 mL, maximum operating pressure 700 bar), separator with cooling jacket (internal volume 200 mL and maximum operating pressure 250 bar), pressure control valve, temperature regulation system, and regulation valves. 

All extractions were performed at pressure 200 bar and temperature 40 °C using 30 g of plant material. The extractions were done with previously pretreated material. Additionally, the control extraction was performed using the plant material without pretreatment. The extraction kinetics was monitored during the extraction time intervals (0.5, 1, 1.5, 2, 3, and 4 h). The pressure and temperature in the separator were 15 bar and 23 °C, while CO_2_ flow (0.3 kg/h) and extraction time (4 h) were constant. Obtained extracts were placed in glass bottles and stored at 4 °C prior to further analysis. Each extraction was performed in triplicate. 

### 3.4. Gas Chromatography-Mass Spectrometry (GC-MS) Analysis

Agilent 8890 gas chromatograph (Agilent Technologies, Palo Alto, CA, USA) connected to mass spectrometer (series 5977E, Agilent Technologies, Palo Alto, CA, USA) was used for the analysis. The components of *O. vulgare* lipophilic extracts were separated on an HP-5MS capillary column (30 m × 0.25 mm, 0.25 μm, Agilent Technologies, Palo Alto, CA, USA). The injector temperature was set at 250 °C with the injected sample of 3 μL in split mode of 1:50. Helium of 99.99% purity was used as a carrier gas in a constant flow regime of 1 mL/min. The following temperature program was set: 70 °C temperature (2 min) which was increased by 3 °C/min to reach 200 °C and maintained at constant temperature for 15 min. The separated components were analyzed with a mass spectrometry (70 eV) with a scanning *m*/*z* range of 30–300. The injector and detector temperatures were 250 °C and 300 °C. Qualitative identifications of the compounds were performed using Wiley 9 (Wiley, New York, NY, USA) and NIST 17 (National Institute of Standards and Technology, Gaithersburg, MD, USA) mass spectral libraries as well as the literature data of retention indices calculated with C_9_-C_25_ alkanes. The analysis of each sample was performed in three replicates and the results are expressed as mean data.

### 3.5. Determination of Antioxidant Activity

The antioxidant activity of extracts was analyzed using the DPPH assay [74]. Different volumes of extracts were mixed with 95% methanol solution and 90 μM DPPH solution. After the 60-min incubation period at room temperature, absorption was measured at a wavelength of 515 nm. The antioxidant activity was expressed as IC_50_ value which represents the concentration of the extract which inhibits 50% DPPH radicals. All the measurements were performed in triplicate.

### 3.6. Statistical Analysis

All analyses were carried out in triplicate and the mean values were considered significantly different at *p* < 0.05 confidence level, after the performance of the one-way ANOVA statistical analysis followed by Tukey test.

## 4. Conclusions

Oregano extracts and their components represent important products that are applied in traditional and modern medicine, as well as in the production of pharmaceutical, cosmetic, food, and insecticides products. Keeping in mind the importance of oregano and the necessity for the implementation of green production principles, the improvement of the processes for obtaining oregano products is a required step. 

Both microwave and enzymatic pretreatments provided intensification of the mass transfer and improvement of the extraction yield and quality of the obtained extracts. In terms of the feasibility of the process, the advantage of microwave pretreatment is that the generated energy is distributed to the material directly, thus high efficiency is achieved in a very short time. The enzymatic pretreatment exhibited an additional benefit because it is a process where there is no increase in temperature over time, but a longer time is required for the pretreatment. The application of microwave radiation (360 W) for 2 min enhanced the extraction yield by more than two-fold and increased the content of oxygenated monoterpenes and the most dominant component, carvacrol. Additionally, the beneficial impact on the total yield and carvacrol abundance was also recorded after the enzymatic pretreatment. In addition, these two treatments led to higher antioxidant properties of the extracts. Considering the possibility of adjusting the selectivity of scCO_2_ by coupling the extraction with the pretreatments, it is possible to increase the selectivity of the process even more and shorten the extraction time. To determine the possibility of industrial scale up, it is necessary to conduct a study on the economic feasibility of the enzymatic and microwave pretreatments which would establish a process with economic benefits.

## Figures and Tables

**Table 1:** GC/MS analysis of oregano extracts obtained by supercritical carbon dioxide extraction (pressure 200 bar, temperature 40 °C, extraction time 4 h).

Compound	RI	Control	ENZP	MWP1	MWP2	MWP3
Monoterpene hydrocarbons						
*α*-Terpinene	1023	0.42	0.29	-	-	-
*p*-Cymene	1032	0.81	0.98	0.36	-	0.55
*γ*-Terpinene	1066	0.35	0.25	0.11	-	0.24
*α*-Terpinolene	1093	0.10	0.12	-	-	-
Oxygenated monoterpenes						
*trans*-Sabinene hydrate	1073	0.26	0.83	0.66	-	0.50
*cis*-Sabinene hydrate	1102	2.49	5.36	4.98	-	5.66
Borneol	1172	0.34	0.38	0.4	-	0.41
Terpinen-4-ol	1182	4.13	3.97	2.49	0.79	2.92
*α*-Terpineol	1195	-	0.79	0.74	0.21	0.92
Thymol methyl ether	1241	0.08	0.07	-	-	-
Carvacrol methyl ether	1250	0.21	0.12	0.19	-	0.31
Thymol	1297	2.47	2.37	2.33	2.26	2.33
Carvacrol	1307	74.89	77.17	80.44	76.94	70.95
2-Methyl-5-(propan-2-ylidene)cyclohexane-1,4-diol	1314	3.19	1.63	2.44	3.50	1.24
Sesquiterpenes						
*trans*-Caryophyllene	1423	0.67	0.75	0.56	1.01	1.06
*β*-Bisabolene	1512	-	-	-	-	0.11
Dihydroactinidiolide	1542	0.14	-	-	-	-
Spathulenol	1581	0.64	0.4	0.48	0.76	0.56
Caryophyllene oxide	1586	-	0.32	0.33	0.74	0.52
Fatty acid methyl ester						
Methyl palmitate	1931	-	-	-	-	0.10
Total peak area (%)		91.19	95.8	96.51	86.21	88.38
Total:		16	17	14	8	16

## Data Availability

Not applicable.
